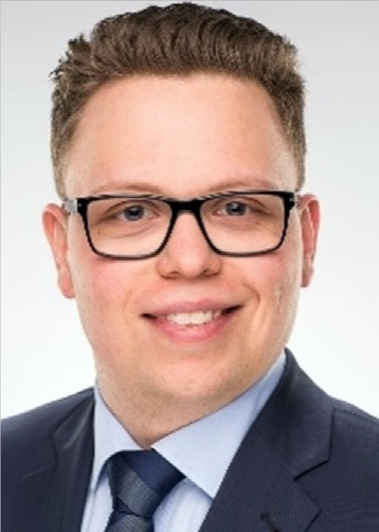# Current Trends in Metal–Organic and Covalent Organic Framework Membrane Materials

**DOI:** 10.1002/anie.202015790

**Published:** 2021-03-03

**Authors:** Bahram Hosseini Monjezi, Ksenia Kutonova, Manuel Tsotsalas, Sebastian Henke, Alexander Knebel

**Affiliations:** ^1^ Institute of Functional Interfaces (IFG) Karlsruhe Institute of Technology (KIT) Hermann-von-Helmholtz-Platz 1 76344 Eggenstein-Leopoldshafen Germany; ^2^ Institute of Organic Chemistry (IOC) Karlsruhe Institute of Technology (KIT) Fritz-Haber-Weg 6 76131 Karlsruhe Germany; ^3^ Department of Chemistry and Chemical Biology TU Dortmund University Otto-Hahn-Str. 6 44227 Dortmund Germany

**Keywords:** covalent organic frameworks, gas separation membranes, metal–organic frameworks, MOF glasses, porous liquids

## Abstract

Metal–organic frameworks (MOFs) and covalent organic frameworks (COFs) have been thoroughly investigated with regards to applications in gas separation membranes in the past years. More recently, new preparation methods for MOFs and COFs as particles and thin‐film membranes, as well as for mixed‐matrix membranes (MMMs) have been developed. We will highlight novel processes and highly functional materials: Zeolitic imidazolate frameworks (ZIFs) can be transformed into glasses and we will give an insight into their use for membranes. In addition, liquids with permanent porosity offer solution processability for the manufacture of extremely potent MMMs. Also, MOF materials influenced by external stimuli give new directions for the enhancement of performance by in situ techniques. Presently, COFs with their large pores are useful in quantum sieving applications, and by exploiting the stacking behavior also molecular sieving COF membranes are possible. Similarly, porous polymers can be constructed using MOF templates, which then find use in gas separation membranes.

## Graphical abstract

Metal–organic frameworks, covalent organic frameworks, polymers, and composites are disruptive key technologies for gas separation membranes. This Minireview highlights the state of the art in materials and material development and their utilization in membrane applications in highly relevant processes such as carbon capture, energy‐ and resource‐efficient separations of basic chemicals, and quantum sieving.

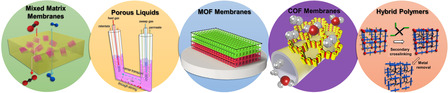

## 1 Introduction

**Figure 1:**
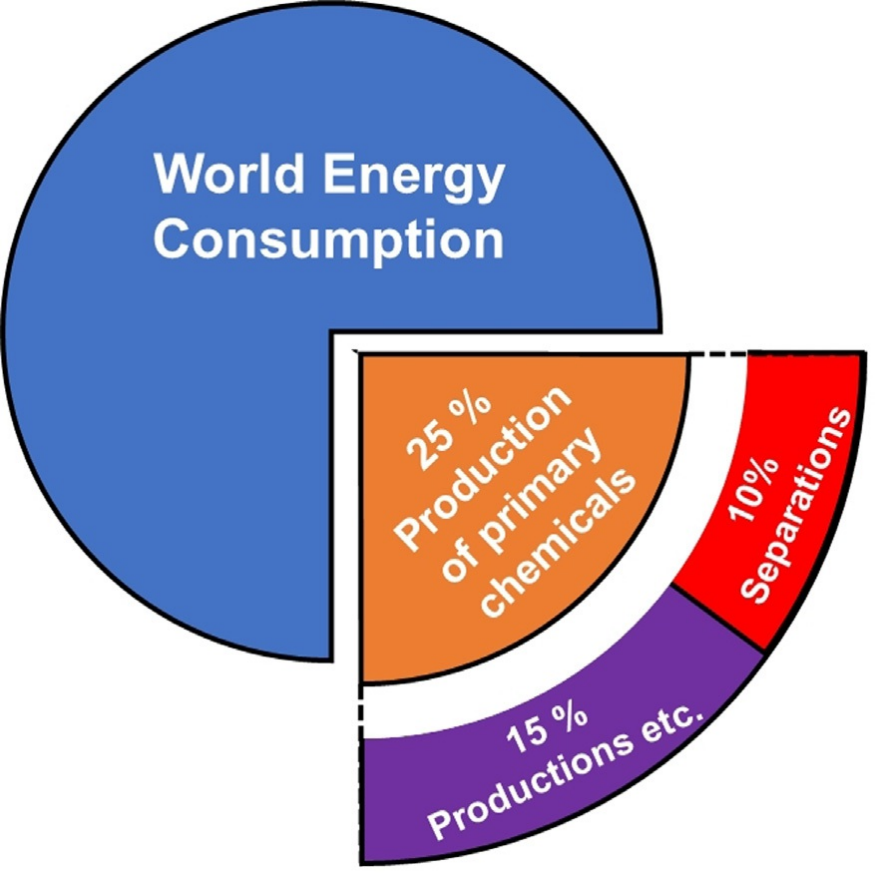
An simplified depiction of the world energy consumption and the amount used only for separation tasks in the production of primary chemicals.[^1^, ^2^, ^3^]

Membranes as a disruptive technology are able to reduce the global energy consumption in the chemical separation of raw materials, as well as actively reduce greenhouse gases actively, and thus form the basis for a sustainable future.[^1^, ^2^] Membrane technology in the petrochemical sector alone could replace distillation processes and save up to 80 % energy in separation processes, which could lead to 8 % savings in the global energy consumption. More than half of the separations are to gas separations (Figure 1).[^1^, ^2^, ^3^] Porous membranes have come a long way from the first description of metal–organic framework (MOF) mixed‐matrix membranes (MMMs) using MOF‐5,^[4]^ the first neat MOF membranes starting with Mn(HCO_2_)_2_ in 2007,^[5]^ and the development of the first ZIF‐8 membranes in 2009,^[6]^ to today's state‐of‐the‐art membranes. Covalent organic frameworks (COF) were used much later for gas separation membranes, since water stability was one of their early issues.[^7^, ^8^] Nevertheless, the first neat and 3D COF membranes comprising COF‐320 date back to 2015,^[9]^ whereas the first experimental CO_2_‐separating MMMs using exfoliated NUS‐2 and NUS‐3 sheets were reported in 2016.^[10]^ When MOF membranes were first developed, the aim was to make these membranes as thick as possible and membranes of 20–300 μm thickness were synthesized. This originated more or less from experience with zeolite membranes, which was drastically changed for thin films.[^6^, ^11^, ^12^] Especially for gas separation and purification, MOFs and COFs show great potential that needs to be unlocked. Today we know that thinner layers are better due to two main factors: higher flux and better selectivity. However, defects are still an issue with thinner films, and single crystals might be considered for recording permeation data. Nevertheless, the goal is downsizing with the preparation of thin films on the nanometer scale and the use of nanoparticles with the best possible polymer–filler interactions in MMMs. Additionally, the development of methods for high reproducibility and control over processes is needed. The aim of this Minireview is to give an overview and highlight trends for the next steps in MOF and COF membrane research, paying particular attention to novel and very hot topics.

The processability of MOF and COF materials is of increasing importance and has caught the attention of the scientific community, leading to many derivative materials with extreme potential, such as porous liquids,[^13^, ^14^, ^15^] amorphous, porous MOF‐based glasses,[^16^, ^17^, ^18^] and porous organic polymers,[^19^, ^20^, ^21^] bringing MOFs/COFs up to the next level. Also, from a more fundamental point of view it is important to step away from the random testing of materials. We will show published data that leads to a deeper understanding of the materials properties, from experiment and theory.[^22^, ^23^] To actually find experimental model systems for membrane separation, single‐crystal permeation testing is necessary.[^24^, ^25^] Also we will address stimuli‐responsive MOF materials, where gas transport has been followed and in situ and framework effects such as gate‐opening, vibrational modes,^[26]^ and electrostatic interactions between guests, linkers, and metal centers could be investigated.^[27]^

For real‐life applications, good processability and performance of MOF and COF membranes is crucial, which is more a matter of post‐processing rather than the original material. Park et al. recently published a paper where they show that material development is the most important step towards good performing membranes.^[28]^ Making new materials out of existing ones by novel processing methods leads to advanced materials.^[29]^ Advanced separation techniques and devices will be highlighted here as well, such as quantum sieving with MOF and COF membranes for isotope separation.[^30^, ^31^] Separation processes are among the greatest challenges worldwide and using membranes could help save the planet^[2]^ by reducing greenhouse gas emission, either actively in CO_2_ separations, or passively by saving energy.

## 2 Advanced MOF Materials for Mixed‐Matrix Membranes

A MMM is made by mixing MOF or COF particles into a polymer matrix and processing the solution into a film or fiber. Making polymer‐filler MMMs from MOF particles is in general simple and cheap.^[32]^ Since most COFs grow to form sheet‐like structures anyway, we will dive deeper into MOFs here, where obtaining sheet‐like particles is not so trivial. Owing to the high structural variety that is offered by the chemistry of MOF materials, special techniques are needed to prepare sheet‐like particles.

### 2.1 Sheet‐Like MOF Particles

MOF nanosheets in the production of ultrathin MMMs in general lead to high performance in separation applications. An alignment in thin polymer composite films is guaranteed due to the shearing forces from the casting approach, making sheet‐like particles extremely interesting for polymer composite films.

**Figure 2:**
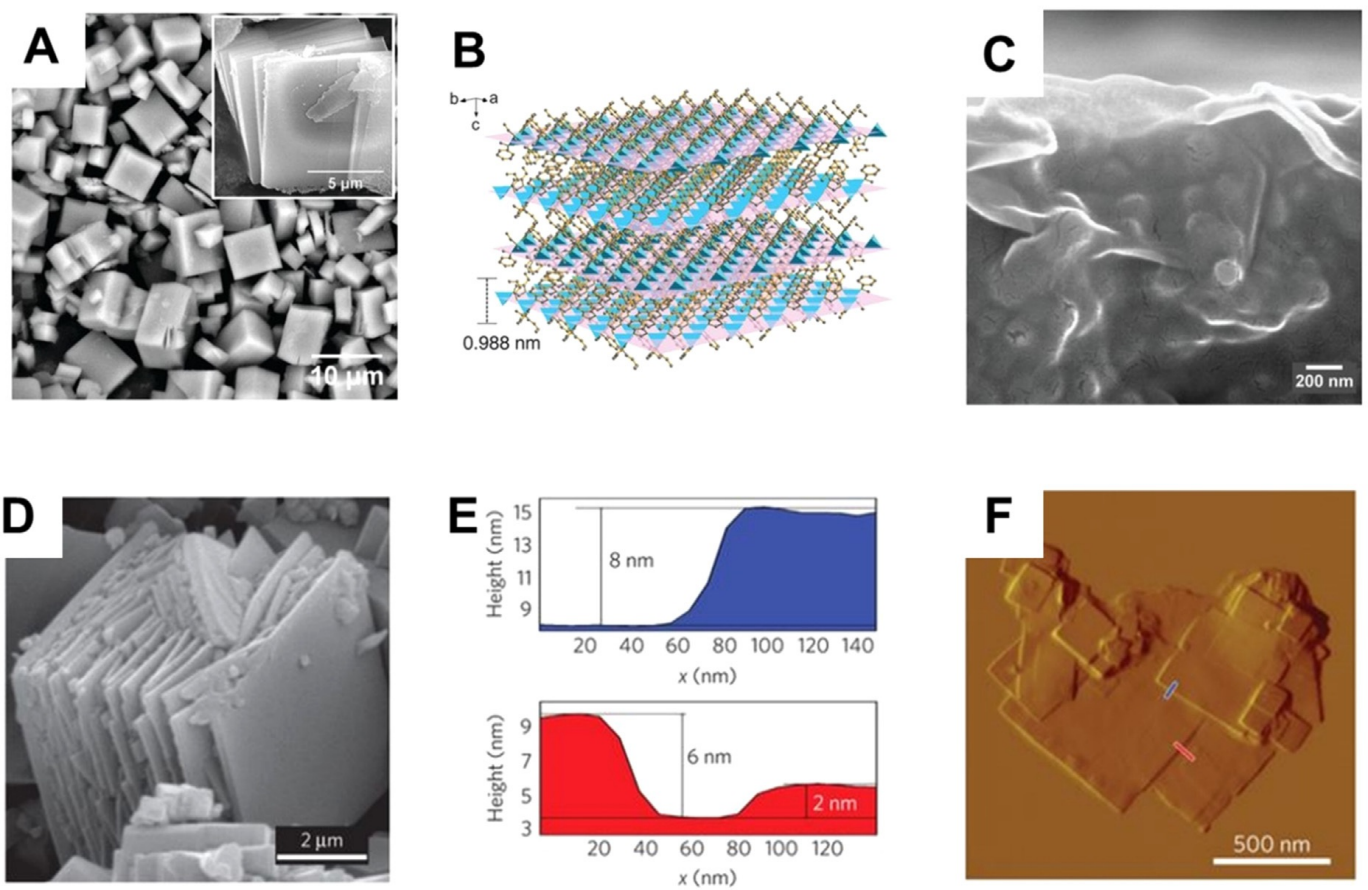
A) SEM image and B) crystal structure of Zn_2_(bIm)_4_; C) SEM image of Zn_2_(bIm)_4_ sheets deposited by filtration as a thin membrane film. From Y. Peng et al.^[33]^ reprinted with permission from AAAS ©2014. D) SEM image showing the layered morphology of CuBDC. E, F) AFM analysis of delaminated CuBDC sheets. Reprinted from T. Rodenas et al.^[35]^ with permission from Springer Nature ©2014.

A very interesting example for the preparation of sheets was published by Peng et al. in 2014,^[33]^ where the lamellar structure of Zn_2_(bIm)_4_ allows the soft physical exfoliation of sheets by wet ball‐milling and mild chemical delamination (Figure 2 A–C). In addition to the use of particles for MMMs, they used a filtration technique to deposit the MOF particles as an ultrathin film on a very rough, porous Al_2_O_3_ support (Figure 2 C). Achieving a layer of 5 nm thickness on a support with that amount of roughness is not possible by solvothermal growth methods or layer‐by‐layer deposition. A solvothermal growth technique will always result in a greater thickness to form a dense and gas‐separating layer.^[33]^

Many lamellar growing crystals can be exfoliated chemically, which was also shown by Pustovarenko et al. in 2018.^[34]^ They used a surfactant‐assisted approach in the synthesis of nanosheets. The first solution contains Al(NO)_3_⋅9 H_2_O and the surfactant hexadecyltrimethylammonium bromide (CTAB); the other solution contains the deprotonated 1,4‐benzodicarboxylic acid (BDC) linker together with 2‐aminoterephtalic acid (2‐ATA) as a promoter. After both solutions are heated, nucleation is induced by blending them. The CTAB forms a lamellar phase and the MOF grows as approximately 100 nm × 100 nm sheets between the surfactant lamellas.^[34]^

Another approach is diffusion‐mediated synthesis at a two‐phase interface, as reported by Rodenas et al.^[35]^ (Figure 2 D–F). MOF‐2(Cu), which already grows as a lamellar MOF, is synthesized at a two‐liquid interface. By diffusion control, the sheets grow along the polar/nonpolar interface. AFM (atom force microscopy) analysis proves to be a lot more accurate than SEM (scanning electron microscopy) imaging for determining sheet thickness.^[35]^

We highlight the production of MOF sheets here, since the morphology has a strong impact on the gas‐separation performance of the membrane. Kang et al.^[36]^ report big differences for [Cu_2_(ndc)_2_(dabco)]_*n*_ in bulk, cubic, and sheet‐like morphologies. They evaluated the different morphologies by the MMM performance in precombustion hydrogen separation and find that 1) downsizing to nanocrystals increases the performance drastically, whereas 2) the use of nanosheets increases the performance further and leads to benchmark performances.[^36^, ^37^] Also the Tsapatsis group reported a strong increase in the selectivity and permeability by almost 70 % when sheet‐like particles are used. Their approach is to directly synthesize Cu(BDC) nanosheets 2.5 μm in length and/or width and only 25 nm thickness. For CO_2_/CH_4_ separation, they find higher values with nanosheets than with spherical particles; they also predicted performances theoretically and came to the same conclusion.^[38]^ A rather important aspect of the incorporation of MOFs into MMMs is the compatibility of filler and polymer.[^38^, ^39^] The implementation of simulations could be a good hint towards defect density in MOF membranes[^38^, ^40^] as it is already widely used to predict the best polymer–filler pairs.^[41]^

### 2.2 Preparation of Mixed‐Matrix Membranes

In addition to material choice and particle preparation, a good procedure for MMM preparation is necessary to achieve the best possible performance. However, gaining the optimal interaction between inorganic fillers and polymers is challenging.

**Figure 3:**
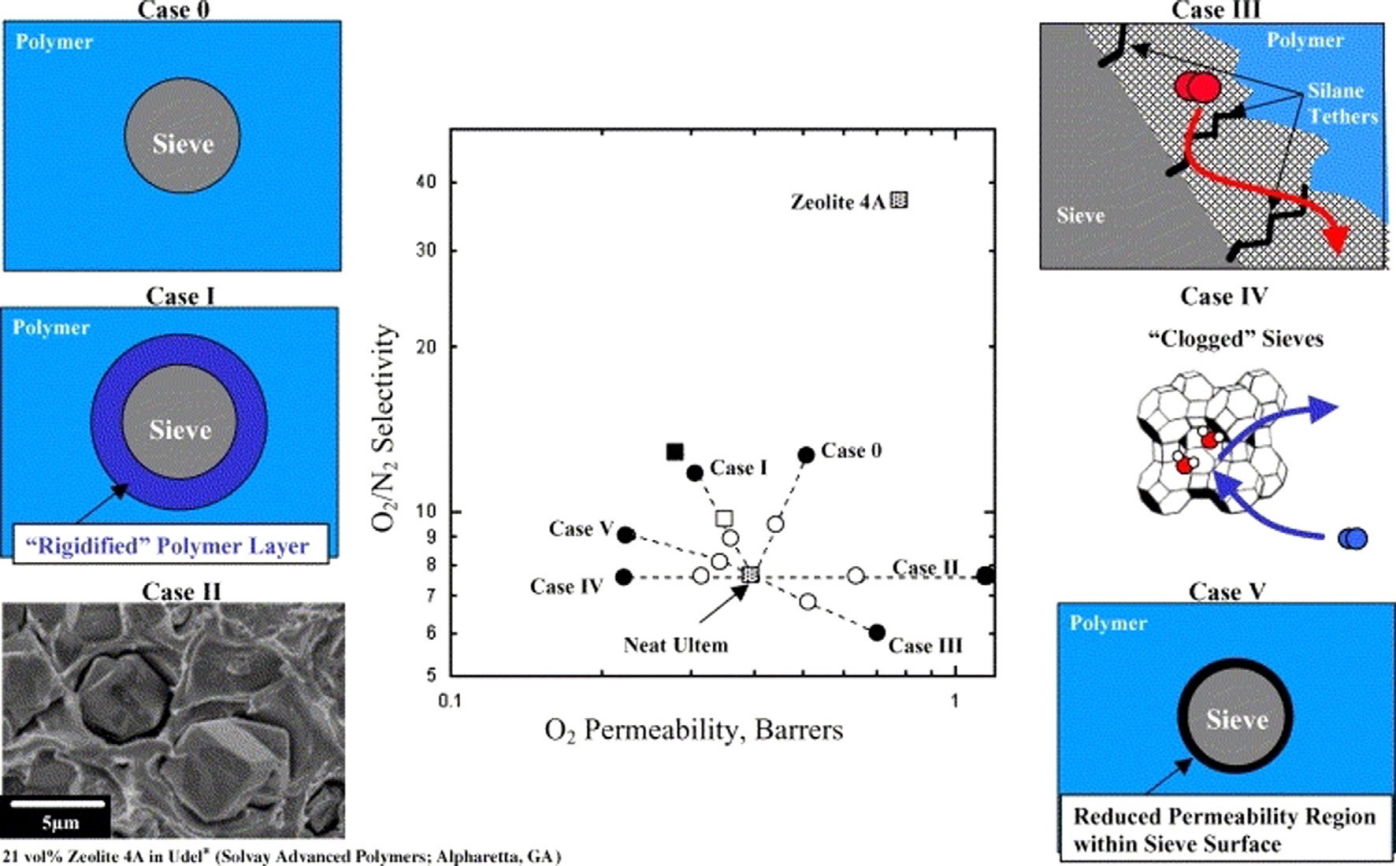
Non‐ideal effects in an MMM lead to a drastic change in performance. Case 0: The ideal case—selectivity and permeability increase. Case 1: A rigidified polymer layer around the filler. Case II: Voids form around the filler, gas breaks through. Case III: “Halo” defects accelerate transport through polymer without transport through the filler. Case IV: Clogged pores exclude transport through the sieve. Case V: Formation of a region with reduced permeability. Reprinted from Moore and Koros^[42]^ with permission from Elsevier ©2012.

The adhesion between polymer and filler materials is strongly dependent on the ratio of inorganic to organic components in the MOF material. For instance, the MIL‐96 material with a very high amount of inorganic Al‐μ_3_‐oxo‐centered trinuclear clusters shows a very poor polymer–filler interaction; it forms agglomerates and even shows crystal ripening in operando, leading to void formation.^[39]^ in their paper on zeolite 4A, Moore and Koros^[42]^ reported several cases of membrane defects that can occur as a result of non‐ideal effects (Figure 3).^[42]^

The distribution of the MOF filler also plays a critical role, because the formation of percolation defects can occur, as shown by Castro‐Muñoz et al.^[43]^ Small defects can have a huge impact and MMM procedures should aim for the perfect embedding of fillers (Case 0) by improving the polymer–filler interaction. Even tiny problems in the compatibility of the components can lead to cracks and defects in the resulting composite membranes, especially when a high content of filler is used.^[44]^ We think that several factors play a role when a poorly performing MMM results: 1) The solvent used for the polymer does not give stable MOF dispersion. This leads to low MOF loading capacity and bad performance due to agglomeration.^[39]^ 2) The proportion of inorganic buildings units and organic linkers is suboptimal.^[42]^ 3) A procedure for good polymer compatibility is not followed. Some recipes consist of complicated mixing procedures, such as the stepwise addition of specific small amounts of the polymer to the colloidal solution to form a stabilizing polymer shell surrounding the nanoparticles.^[45]^

### 2.3 Porous Liquids for Liquid Processing of MMMs

**Figure 4:**
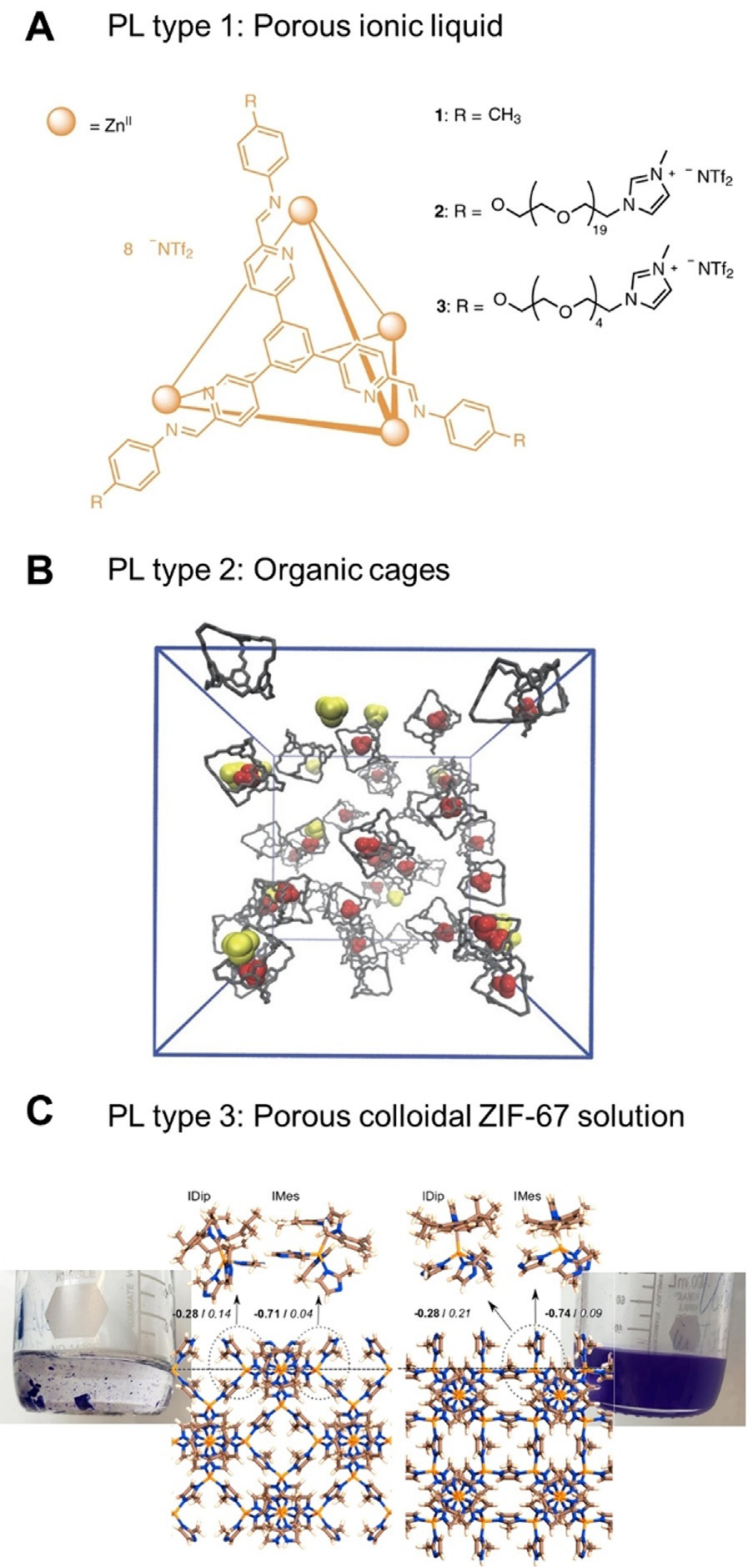
A) PL Type 1: Polyether‐functionalized cage framework acting as an ionic liquid. Reprinted from Ma et al.^[49]^ with permission from Springer Nature ©2020. B) PL Type 2: Organic cages with permanent porosity in the crown ether. Reprinted from Giri et al.^[15]^ with permission from Springer Nature ©2015. C) PL Type 3: Colloidally disperse, NHC‐functionalized ZIF‐67 in the non‐penetrating solvent mesitylene. Reprinted from Knebel et al.^[55]^ with permission from Springer Nature © 2020.

Porous liquids (PLs) are a novel class of porous materials that have been known for only a few years. First proposed by the James group in 2007,^[46]^ they reported the experimental breakthrough in 2015.^[15]^ PLs are materials with a special feature: porous cage structures with a maximum pore diameter smaller than that of the solvent molecules surrounding them. Thus, they remain empty and accessible for gases while in the liquid state.^[47]^ PLs can be categorized in three different types[^46^, ^48^] (Figure 4): Type 1 PLs are cage materials that are liquid by themselves. The only example known to us thus far are polyether‐functionalized coordination cages that act as ionic liquids.^[49]^ Carefully said, MOF‐based melts, for example, made of ZIF‐62 might also be regarded as type 1 porous liquids (see below).[^50^, ^51^, ^52^] Type 2 porous liquids are organic cages that can be dissolved in a sterically demanding solvent. For instance, organic cages could be obtained by cycloimination of (15*S*,16*S*)‐1,4,7,10,13‐pentaoxacycloheptadecane‐15,16‐diamine with the cross‐linker benzene‐1,3,5‐trialdehyde. Here, 15‐crown‐5 serves as the solvent for the cages.^[15]^ Organic cages were recently used for propylene/propane separation with great results.^[53]^

In contrast, a Type 3 PL, is a colloidal solution of solid framework particles. There are reports of ZIF‐8 and zeolite ZSM‐5 dispersed in ionic liquids that are Type 3 PLs.[^13^, ^54^] A new finding also suggests MOFs and zeolites dissolved in long‐chain organic oils and silicon oils yield Type 3 PLs.^[55]^

MOF‐based PLs are able to load MMMs with a high wt. % due to high colloidal stability. Recently, ZIF‐67 and ZIF‐8 nanoparticles could be functionalized on their outer surface by N‐heterocyclic carbenes (NHCs) to make them solution processable. The NHC‐functionalized ZIFs were able to form monodisperse, highly stable colloidal solutions in nonpolar solvents, in which many polymers are prepared. Using ZIF‐67 and ZIF‐8 with NHC functionalization also leads to a very good interaction with the polymer matrix (6FDA‐DHTM‐Durene and 6FDA‐DAM), enabling very high MOF loadings of up to 47.5 wt. % inside the polymer, while also being able to separate gases in the liquid state.^[56]^

## 3 MOF Glasses for Membranes

**Figure 5:**
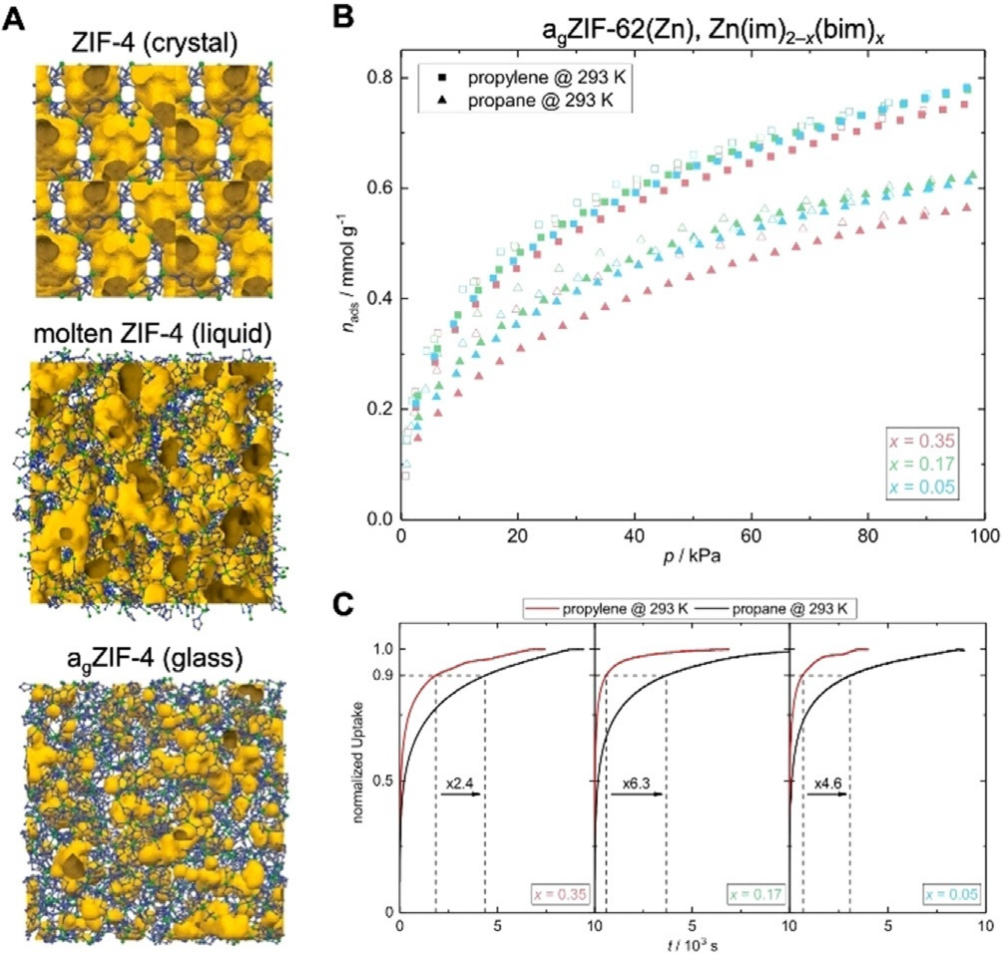
A) Atomic configuration of the crystalline, molten, and glass phases of ZIF‐4; C (gray), N (green) Zn( blue), open void space (yellow). Reprinted from Gaillac et al.^[52]^ with permission from Springer Nature ©2017. B) C_3_H_6_ and C_3_H_8_ sorption isotherms of a_g_ZIF‐62 glasses containing various amounts (*x*) of the bim^−^ linker. C) Kinetic sorption profiles for C_3_H_6_ and C_3_H_8_. Reprinted from Frentzel‐Beyme et al.^[59]^ with permission from the American Chemical Society ©2019.

As already mentioned, some MOFs in the ZIF family melt and form stable liquids, when heated under inert atmosphere (typically Ar or N_2_).[^17^, ^18^] The inert atmosphere is crucial in order to prevent thermal oxidation and decomposition of the ZIF melt. A prototypical example is ZIF‐4, which melts at ≈590 °C before thermal decomposition at ≈600 °C (Figure 5 A).^[17]^ Molecular dynamics simulations on the thermal behavior of ZIF‐4 yielded further insights into the process of MOF melting. The Zn−N bonds dissociate on the ps timescale, generating undercoordinated Zn^2+^ cations.^[52]^ Subsequently, new Zn−N bonds are formed by association of other imidazolate linkers. These simulations suggest that the liquid ZIF‐4 still possesses microporosity similar to the crystalline phase, but there is currently no experimental proof that the pores in liquid ZIF‐4 are accessible. Nevertheless, the liquid ZIF‐4 can be regarded as a variation of a Type I PL. Quenching the liquid ZIF‐4 to room temperature generates a glass denoted a_g_ZIF‐4 (a_g_=amorphous glass).[^17^, ^18^] The glass features a frozen atomic configuration of the supercooled liquid state. X‐ray total scattering experiments show that the glass is amorphous (i.e. does not possess long‐range order), but it possesses a local structure that is identical to that of the crystalline ZIF.[^17^, ^18^, ^57^]

In the past few years, a number of other (mixed‐linker) ZIFs have also been shown to melt and form glasses.[^18^, ^50^, ^51^, ^58^] Prominent examples include ZIF‐62 and TIF‐4, which are structurally closely related to the prototypical ZIF‐4 and feature the same **cag** network topology, but a secondary imidazolate linker. Importantly, these mixed‐linker ZIFs generally feature a much lower melting point than conventional ZIF‐4. As demonstrated for ZIF‐62, the melting point of the crystals, as well as the glass transition temperature of the corresponding glasses, can be adjusted precisely by the amount of secondary linker, resulting in a melting point of only ≈372 °C, more than 200 °C lower than that of ZIF‐4.^[59]^ Most importantly, mixed‐linker ZIF glasses feature permanent porosity for a variety of gases, such as CO_2_, H_2_, and several hydrocarbons (Figure 5 B).[^50^, ^51^, ^59^, ^60^] Even though the sorption capacity of the ZIF glasses is typically approximately 50 % lower than the capacity of their crystalline parent frameworks, this finding sets the stage for the application of glassy ZIFs in gas separation. Kinetic sorption measurements of propane and propylene in a_g_ZIF‐62 materials showed that propylene is adsorbed much faster than propane, demonstrating the potential of ZIF glasses for gas separation applications (Figure 5 C).^[59]^

As an important consequence of their liquid‐state processability,^[61]^ ZIF glasses can easily form composites with other materials. Bennett and co‐workers prepared MOF‐crystal‐glass composites of crystalline MIL‐53 in a matrix of a_g_ZIF‐62.^[62]^

**Figure 6:**
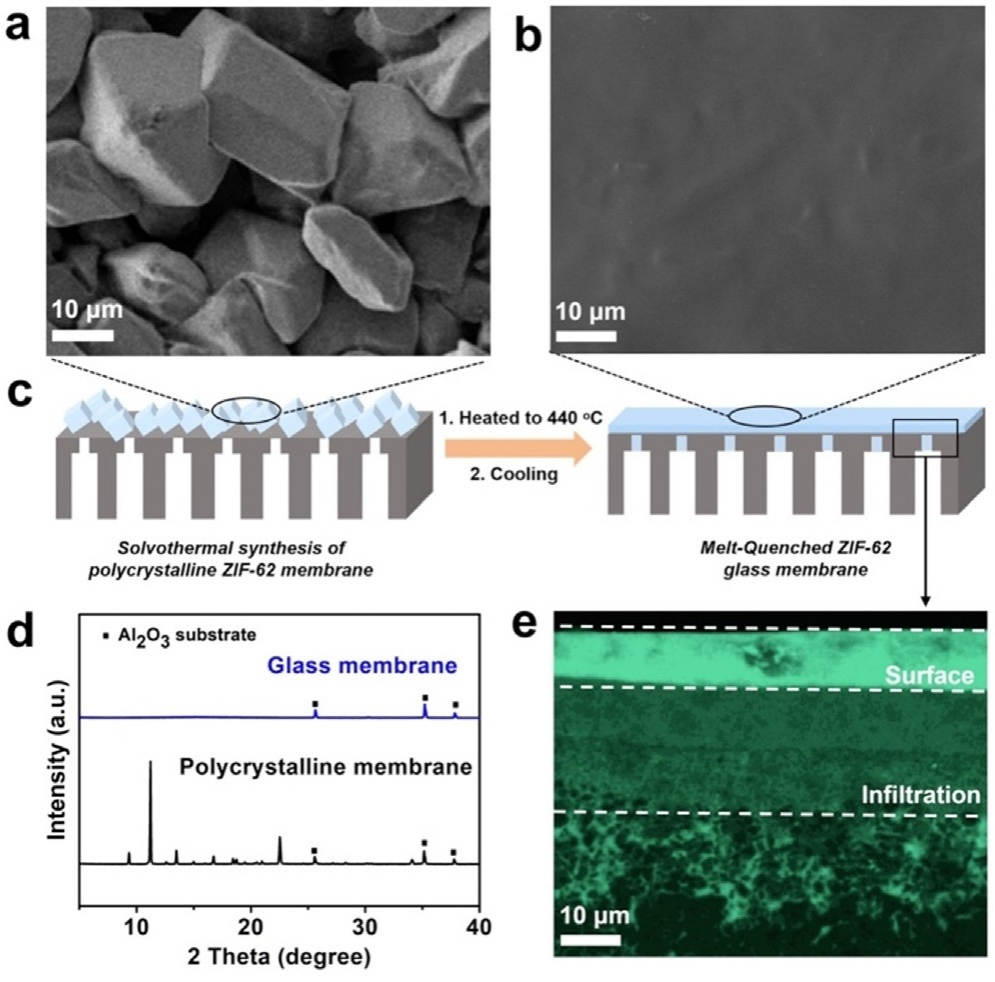
SEM top view of a) polycrystalline ZIF‐62 membrane b) a_g_ZIF‐62 glass membrane. c) Formation of the ZIF glass on the α‐Al_2_O_3_ substrate. d) XRD patterns of the ZIF‐62 and a_g_ZIF‐62 membranes. e) Cross‐section of the glass membrane. Energy‐dispersive X‐ray mapping shows Zn distribution (green). Reprinted from Wang et al.^[63]^ with permission from Wiley‐VCH ©2020.

When it comes to membrane applications, MOF glasses have two conceptual advantages: 1) The glasses can be easily processed and deposited in their liquid state and 2) there are no grains or grain boundaries in the isotropic glass. Grain boundaries are unavoidable in polycrystalline MOF membranes and represent a fundamental problem, since these boundaries represent defects or microscopic cracks that can significantly compromise the selectivity of the membrane. Wang and Jin et al. reported the first ZIF glass membrane made of an a_g_ZIF‐62 film on a porous α‐Al_2_O_3_ support (Figure 6).^[63]^ In analogy to the ZIF bulk glasses, the ZIF glass membrane was prepared by melt‐quenching a solvothermally synthesized polycrystalline ZIF‐62 film (thickness ≈70 μm) on a α‐Al_2_O_3_ support under inert atmosphere. The original ZIF‐62 film featured intergrown microcrystals associated with gaps, pinholes, and grain boundaries. The melt‐quenched a_g_ZIF‐62 membrane is smooth and defect‐free without any grain structure (Figure 6). The ZIF‐62 glass membrane showed enhanced gas separation properties, with separation factors of 50.7 (H_2_/CH_4_), 34.5 (CO_2_/N_2_), and 36.6 (CO_2_/CH_4_).^[63]^

Another recent proof‐of‐concept study reported an MMM consisting of a_g_ZIF‐62 imbedded in a polyimide matrix.^[64]^ A ZIF‐62/polyimide‐MMM containing 20 wt. % ZIF‐62 showed an improvement of its CO_2_/N_2_ selectivity by ≈27 % upon thermal transformation of the crystalline ZIF‐62 to a_g_ZIF‐62 at 440 °C.

## 4 Neat MOF Membranes

**Figure 7:**
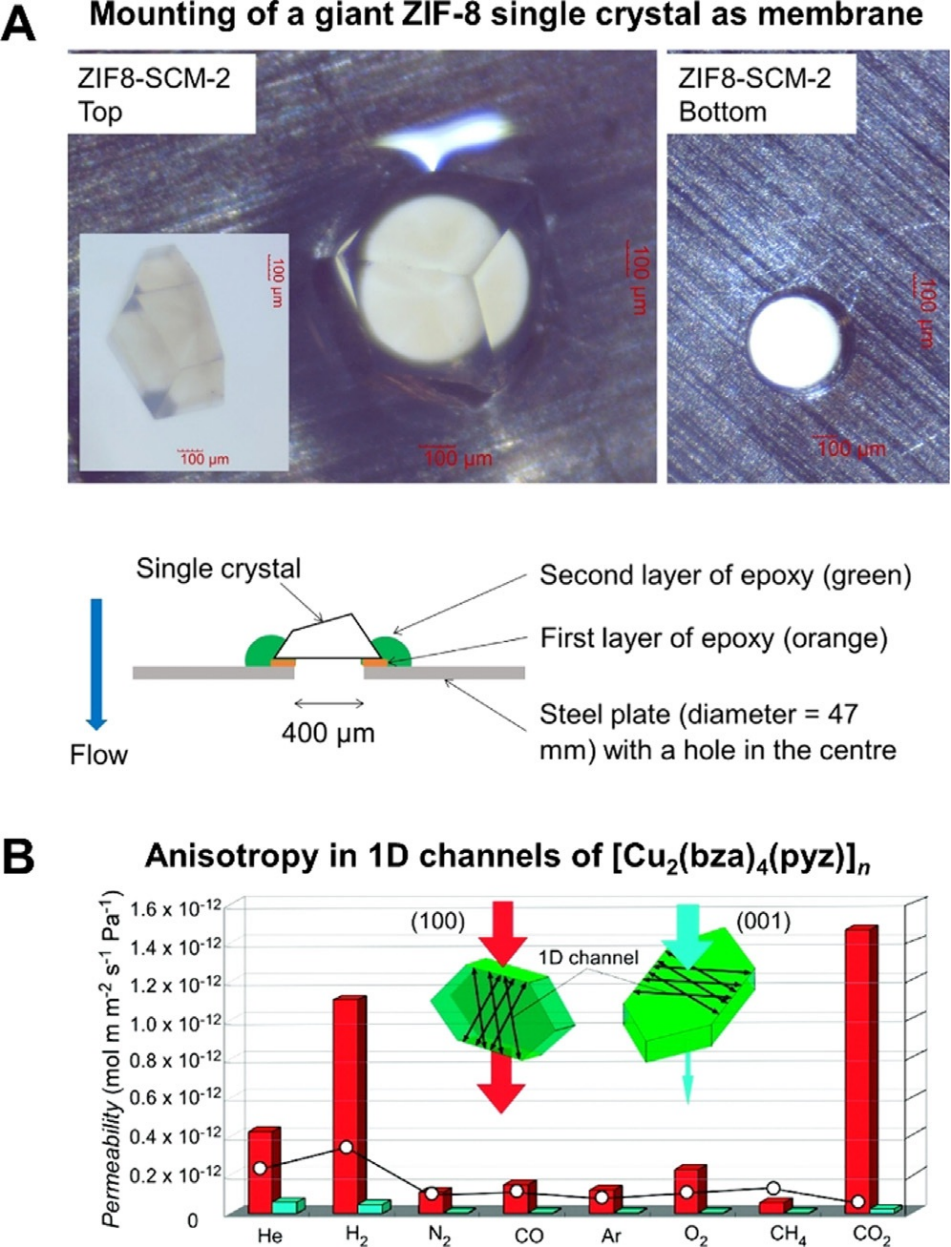
A) ZIF‐8 single crystal mounted on a metal plate with a 100 μm hole in it. Reprinted from Chen et al.^[25]^ with permission from Elsevier ©2019. B) Measuring anisotropic gas permeation through [Cu_2_(bza)_4_(pyz)]_*n*_ with 1D channels. The channels in the (100) direction are free for gas transport, no gas permeation occurs in the (001) direction. Reprinted from Takamizawa et al.^[65]^ with permission from the American Chemical Society ©2010.

Neat MOF membranes are usually grown on ceramic supports by solvothermal methods. Since ceramic membranes are 1000 times more expensive (per m^2^) than polymeric films, neat MOF membranes are hard to apply in industrial settings.^[11]^ MOF membranes on ceramic supports cannot be used for antifouling treatment such as decoking, since MOFs would burn as well—a huge disadvantage. The one‐time use of these membranes would be a huge cost factor. Nevertheless, MOF membranes have long been synthesized on ceramic supports, and we do not want to exclude potential applications. From a fundamental perspective, especially for understanding the transport properties of the MOF itself, it is of great importance to produce and measure crystalline intergrown layers. However, of particularly interest is the gathering of “true permeation data” from single‐crystalline membranes (Figure 7).[^25^, ^65^] The “true” separation properties can be measured using single crystals, and diffusion constants and real permeation data can be obtained.^[66]^ Although single‐crystalline membranes would be the ultimate goal of membrane science, they cannot be obtained on a large scale. Therefore, the layer‐by‐layer growth of surface‐anchored metal–organic frameworks (SURMOFs) could be a key approach, because it produces an almost perfect layer. This technique offers large‐scale processability of neat MOF layers with highly defined thickness.^[67]^ The crystallinity can be so high that HKUST‐1 films become transparent, since the characteristic blue color centers are missing.^[68]^ It has been demonstrated that this technique also offers applications for neat MOF membranes, with the first example being ZIF‐8.^[69]^ The defined heteroepitaxial growth of ZIF‐67 and ZIF‐8 with exactly the same layer height has been shown.^[70]^ The technique's only current drawback is the limited number of accessible MOF structures, due to the solvent and temperature limitations of the method. Recently, UiO‐66‐NH_2_ has been made available by liquid‐phase epitaxy^[71]^ and many more complicated frameworks will be available as SURMOFs in the near future. The SURMOF technology will set standards as a tool since it is possible to follow the exact growth of neat MOFs step by step.^[72]^

### 4.1 Stimuli‐Responsive Neat MOF Membranes

Whereas the response of MOFs to applied pressure and temperature is a well‐known concept,^[73]^ MOFs can also respond to other types of external stimuli, such as light and electric fields.^[74]^ The first conceptional proof of electric‐field‐stimulated MOFs was shown theoretically by the group of Maurin et al. for the breathing behavior of MIL‐53.^[75]^ In general, electric fields should be able to align dipolar moments inside the MOF structures (e.g. the linker molecules).^[76]^ There are theoretical concepts that use strongly dipolar linkers to align dipolar moments in a high‐voltage electric field.^[77]^ Nevertheless, for MOFs that are feasible for membranes or adsorptive separation techniques (ZIF‐8, MIL‐53 etc.), theory and practical work conclude that electric fields must exceed the breakthrough voltage before linker orientation occurs.[^75^, ^76^, ^78^] Nevertheless, some MOFs are known for ferroelectric effects, even when it is nearly impossible to measure the hysteresis at accessible temperatures.^[79]^ This is the case for ZIF‐8 with the space group of *I* $\bar{4}$ 3*m*, which is able to display a structural transformation inside an electric field (500 V mm^−1^). The symmetry is reduced to the monoclinic space group *Cm* and the symmetry switches further to *R*3*m* at higher fields. As consequence of the *E*‐field‐driven transformation, a change in rotational energy barriers and a higher framework stiffness arises, which increases molecular sieving.^[78]^ Zhou et al. demonstrated the direct synthesis of a ZIF‐8 membrane in an electric field. There, ZIF‐8 crystallizes directly in the *Cm* space group during the growth process. This leads to a very good molecular‐sieving ZIF‐8(*Cm*) membrane that outperforms the usual ZIF‐8(*I* $\bar{4}$ 3*m*) membrane.^[80]^

Utilizing light‐responsive molecules inside the pores of MOFs, either in the backbone or as a guest molecule, leads to controllable gas transport and adsorption.^[27]^ When, for example, azobenzene (AZB) is introduced in the pores as guest molecules, an old concept already used in zeolites,^[81]^ molecular transport through the pores can be influenced by gating effects, as concluded from in situ gas permeation results.^[82]^ Another case shows AZB molecules as side chains on the backbone of MOFs, leading to adsorptive separation differences due to *cis–trans* isomerization, which effects adsorption of CO_2_ by hindering its diffusion via interaction with its quadrupolar moment.[^27^, ^83^] Light response has also been shown for MMMs, where JUC‐6 and PCN‐250 were used inside a Matrimid® 5218 membrane. Nevertheless, thermal effects in the MMM could also have an effect on the gas separation.^[84]^ The field of stimuli‐responsive membranes and switches^[85]^ is strongly growing and the visionary aim towards a universal membrane system for all different kind of separations looks achievable.

## 5 Membranes Based on Covalent Organic Frameworks

The first COFs reported by Yaghi et al. in 2005^[8]^ were constructed utilizing the reversible reaction of boronic acid trimerization to form boroxine COF (e.g. COF‐1), or their condensation with catechols to form boronate ester COFs (e.g. COF‐5). Both reactions proceed with the evolution of H_2_O and therefore the equilibrium in these reversible reactions is dependent on the water content and humidity stability is limited. Since then, many different reversible reactions have been used for the formation of COFs, also providing high chemical, thermal, and mechanical stabilities. The very first example of a COF studied in terms of gas separation was reported by Zhu et al. in 2013.^[86]^ The microporous boronate ester 3D COF (MCOF‐1) derived from tetra(4‐dihydroxy‐borylphenyl)methane and 1,2,4,5‐tetrahydroxybenzene was synthesized and investigated as an adsorbent for various gases, such as methane, ethylene, ethane, and propane. Although no experimental data was provided in this study, it clearly demonstrated the high potential of COFs for gas separation, and since then research on COF membranes for gas separation has increased drastically.^[86]^

### 5.1 Neat COF Membranes and the Bilayer Approach

Due to their large pore sizes, COFs have found use in gas separation membranes as components of stacked bilayers on porous supports (e.g. porous α‐Al_2_O_3_, cellulose acetate, Nylon). The bilayer approach uses two different materials stacked upon each other. This was realized in both bottom‐up and top‐down ways. The first examples relied on α‐Al_2_O_3_ supports for the synthesis of a μm‐thick film of azine‐linked COF (ACOF‐1) via the condensation of 1,3,5‐triformylbenzene and hydrazine hydrate under solvothermal conditions.^[87]^ The performance of the resulting membranes was measured for CO_2_/CH_4_ mixed‐gas separation using a Wicke–Kallenbach permeation apparatus and reached α(CO_2_/CH_4_)=97.1 under optimized conditions. In ACOF‐1 CO_2_ strongly adsorbs to the polar framework and the permeance of CH_4_ is significantly lowered in the mixture compared to the single‐gas permeance, which was explained by competitive adsorption mechanisms. This is a fine demonstration for the critical need of mixed‐gas permeances as a trustable measure of the material performance. In a double‐layer system, using imine‐linked COF (LZU1) on top of ACOF‐1, performance could be increased.^[88]^

**Figure 8:**
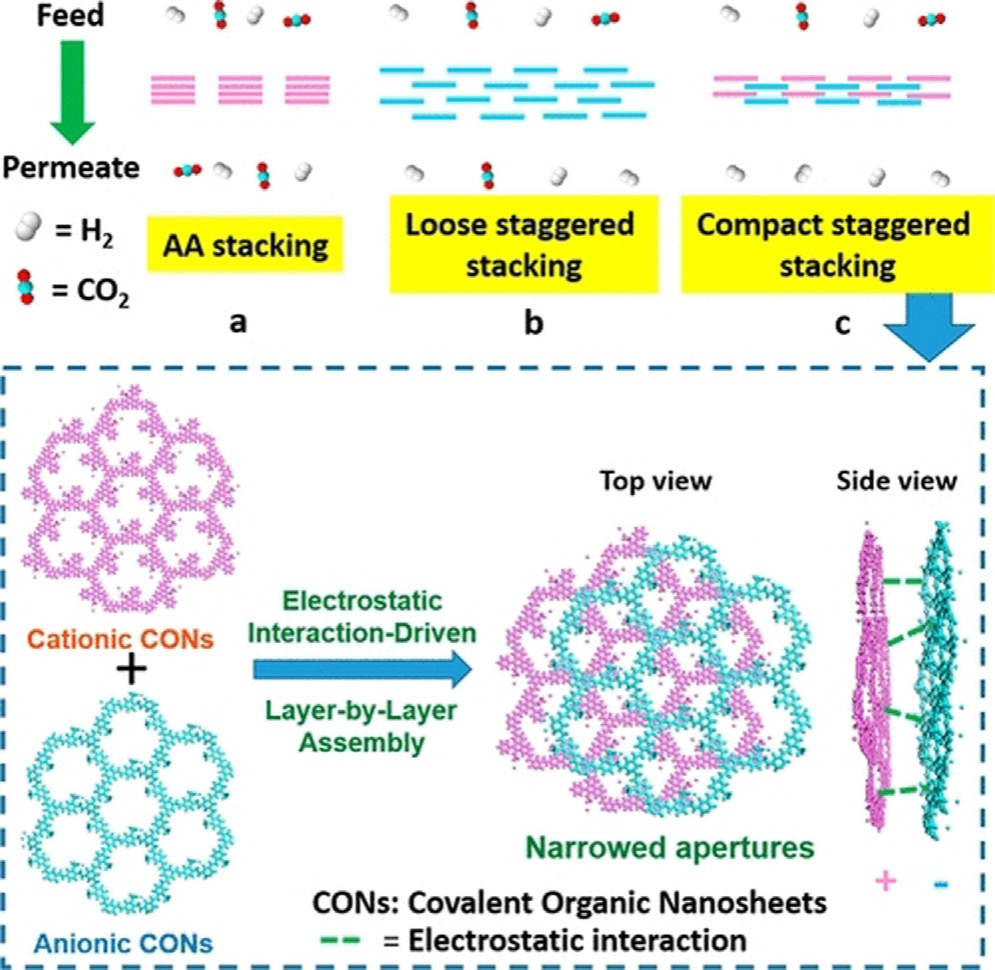
The COF bilayer approach using cationic and anionic COF sheets to prepare staggered bilayer membranes for H_2_/CO_2_ separation. Reprinted from Ying et al.^[89]^ with permission from the American Chemical Society ©2020.

Due to the large pores of COF materials, molecular sieving can be achieved using an interlaced layer (“gate‐closing” approach) between two COFs; outstanding H_2_/CO_2_, H_2_/N_2_, and H_2_/CH_4_ selectivities were achieved for the LZU1/ACOF‐1 bilayer membrane. An elegant way to realize a similar approach was recently reported by the Zhao group for two different COFs—an anionic imine‐based COF, containing sulfonate groups, and a cationic imine‐based COF, containing N‐alkylated phenanthridine bromide (Figure 8). Both were deposited by the Langmuir–Schaefer method as thin films on a porous α‐Al_2_O_3_ support.^[89]^ Strong electrostatic interactions led to the formation of a compact staggered stacked film with narrow pores which could achieve very high H_2_/CO_2_ selectivity.^[89]^ One unique application for COF membranes aims towards the quantum sieving effect for the separation of hydrogen isotopes at cryogenic temperatures.^[30]^ COF‐1, prepared at room temperature in the presence of pyridine, contains one pyridine molecule per boroxine ring, limiting the pore size and provide kinetic hindrance at the aperture at cryogenic temperatures. Separation of an H_2_/D_2_ isotope mixture could be achieved at 26 mbar loading pressure for the temperature range between 20 and 100 K with a selectivity α(D_2_/H_2_) of 9.7±0.9 at *T*_exp_<30 K and 3.1±0.5 at 70 K, exceeding the selectivity of commercial cryogenic distillation processes.

### 5.2 COF‐Based MMMs

In 2016 Zhao^[10]^ and Gascon^[90]^ showed the first COF‐based MMMs in mixed‐gas separation. Exploring different 2D COF materials, the Zhao group used NUS‐2 and NUS‐3, which have the ultimate advantage of high stability against water.^[10]^ These COFs are derived from the condensation of triformylphloroglucinol with hydrazine hydrate (NUS‐2) or 2,5‐diethoxyterephthalohydrazide (NUS‐3). The presence of ‐OH groups allows keto–enol tautomerization to act as a locking mechanism for the labile imine bonds by transferring them into nondynamic and chemically inert β‐ketoenamine bonds. This stable 2D COF could be exfoliated to COF nanosheets and incorporated up to 30 wt. % polyetherimide (Ultem®) and polybenzimidazole (PBI). The mixed‐gas separation performance of these MMMs using an equimolar H_2_/CO_2_ gas mixture was *α*=5.80 for NUS‐2@Ultem and *α*=31.40 for NUS‐2@PBI. The MMM prepared using 2D imine ACOF‐1 in Matrimid® displays a high selectivity for CO_2_/CH_4_ gas mixtures and a twofold increased CO_2_ permeability.^[90]^ Significantly increased CO_2_ permeability owing to electrostatic interactions in COFs seems to be common, since other imine‐based COFs (e.g. those formed by the condensation of melamine and terephthaldehyde) in PIM‐1^[91]^ also showed this effect.

With regards to the polymer–filler interaction described in Section 2.2, purely organic, covalently bonded COFs typically perform very well in MMMs, in contrast to MOFs or zeolites.^[92]^ Nevertheless, the compatibility of COFs and polymers can be further improved. For example, a matrix able to make van der Waals interactions (e.g. hydrogen bonds between COF and polymer chains) allows for better component mixing.[^93^, ^94^, ^95^] When an NH‐rich imine‐COF was combined with an NH‐rich PBI matrix, a COF loading of 50 wt. % was possible,^[93]^ whereas OH‐rich COF‐5 showed good compatibility with PEG‐containing polyether block amide (PEBAX® or VESTAMID® E).^[95]^ The Wang group^[96]^ premodified the surface of 2D imine COF‐LZU1 particles with polyvinylamine chains, which resulted in the good compatibility of the modified COF with polyvinylamine matrix.

## 6 Advanced MOF/Polymer and COF/Polymer Hybrids

MOFs, COFs, and classical polymers feature different and often complementary properties in terms of their stability, surface area, and regular structure, as well as their processability.^[97]^ We want to provide a brief summary of recent approaches going one step further in the combination of MOF/COF and polymers, in which polymer species are inserted inside the MOF or COF pores and serve as precursors for MOF or COF growth, or in which MOFs/COFs template the synthesis of porous polymer networks.^[98]^ These new approaches can lead to improved performance and stability in a variety of membrane or separation applications, including water treatment and gas separation, for example, for CO_2_ sequestering.[^21^, ^99^]

**Figure 9:**
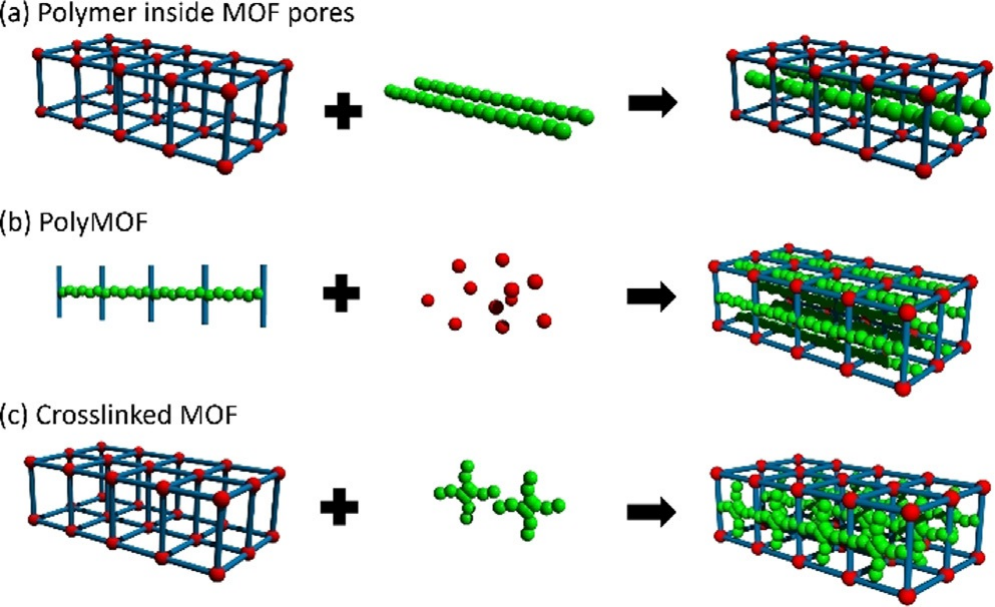
Strategies for the tight integration of MOFs and COFs with polymer materials by a) integrating the polymer chain inside the pores, b) using polymeric linkers as precursors, or c) crosslinking the linker molecules post‐synthetically. Reprinted from Begum et al.^[98]^ with permission from the American Chemical Society ©2020.

The formation of advanced MOF–polymer hybrid devices and membranes can be divided into three main approaches categorized as: a) polymer synthesis within the MOF pores, b) PolyMOFs, and c) crosslinked MOFs (Figure 9).^[19]^

The described approaches intend to either enhance the properties of the MOF or COF membranes by the combination of polymers with enhanced processability and stability, or enhance the performance of polymeric materials by the advantages of MOFs such as their high degree of order across multiple length scales, making it possible to implement high‐throughput computational screening approaches.[^23^, ^100^] We envision that these new concepts in the tight integration of MOF and COF materials on the one hand and polymer materials on the other hand will be further exploited in the future to tackle real‐world separation challenges.

## 7 Perspectives

There is a critical need for disruptive technologies such as membranes to lower the energy use of the chemical industry and reduce greenhouse gas emission worldwide. MOFs and COFs are materials with extraordinary properties to help separations in the petrochemical sector, such as propylene/propane, as well as in direct CO_2_ capture and the sustainable production of CH_4_. To make use of the potentially best materials for these processes, a targeted material development, rather than synthesis of more and more novel materials, is crucial. We have described the non‐ideal polymer–filler effects in MMMs, which are already known but too often neglected.^[42]^ We have recounted many pioneering studies of material development that are highly suitable for membrane science and we encourage people to work on these: porous liquids and the liquid processability of MOF and COF particles in the production of polymer composite membranes; the formation of glasses composed of molecular‐sieving ZIFs, opening up totally new perspectives, such as grain‐boundary‐free films and the production of hollow fiber membranes made of neat MOF‐glass. On the other hand, we think it is a crucial step and the main task of science to do fundamental research, determine material parameters (e.g. using single crystals for diffusion studies) and go to next‐level separations such as quantum sieving. Even though, for some of these processes finding an application is rather challenging, there is a lot to learn fundamentally: As an example, stimuli‐responsive materials taught us a lot about MOFs and gas transport stimulation, whereas applications as “universal” membranes switching to the desired application are yet futuristic. The prerequisite here is that the phenomena be fully understood and that process integration is available for the spectrum of MOF, COF, and polymer materials. A combined theoretical and experimental approach is necessary to develop these materials towards a key technology and transfer them to industry.

## Conflict of interest

The authors declare no conflict of interest.

## Biographical Information

*Bahram Hosseini Monjezi studied Chemistry at the Isfahan University of Technology (Iran) and received his master's degree with Prof. Mehran Ghiaci in 2012. After several years at the Research Institute of Petroleum Industry (Iran), he returned to academia and is currently conducting PhD research in the group of Alexander Knebel at the Karlsruhe Institute of Technology (KIT) at the Institute of Functional Interfaces (IFG) (Germany). His work focuses on the synthesis and properties of porous‐organic/metal–organic framework membranes and thin films*.

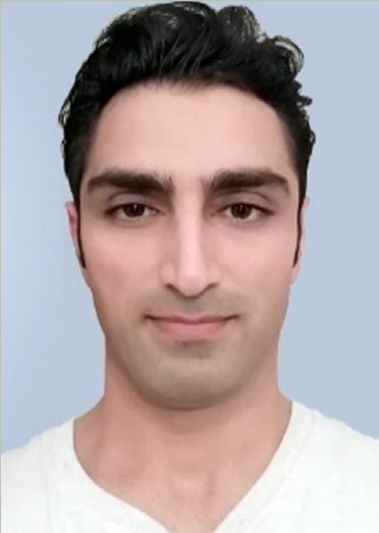

## Biographical Information

*Ksenia Kutonova studied Chemistry and Chemical Engineering at the Tomsk Polytechnic University (Russia) and received her PhD in Organic Chemistry there in 2016. Since 2017 she has conducting postdoctoral research in Prof. Stefan Bräse's group at Karlsruhe Institute of Technology. Her research interests are organic building blocks and synthetic transformations for the preparation of application‐driven materials*.

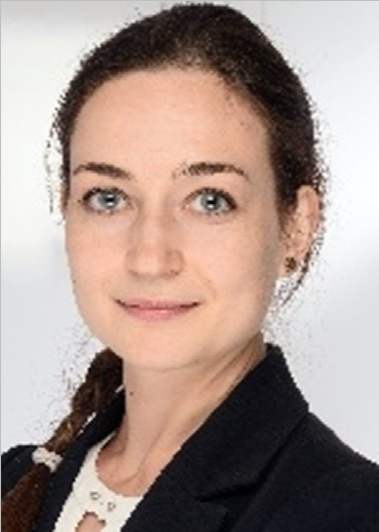

## Biographical Information

*Manuel Tsotsalas studied Chemistry and obtained his PhD in 2010 at the University of Münster (Germany) with research stays at the University of Rennes (France) and Cornell University (USA). After a postdoctoral stay with Susumu Kitagawa and Shuhei Furukawa at Kyoto University (Japan), he moved to the Karlsruhe Institute of Technology, where he obtained his Habilitation in 2019. He currently leads a Helmholtz Young Investigator Group there. His research interests focus porous polymers and their application as novel nanomembranes and bioactive surface coatings*.

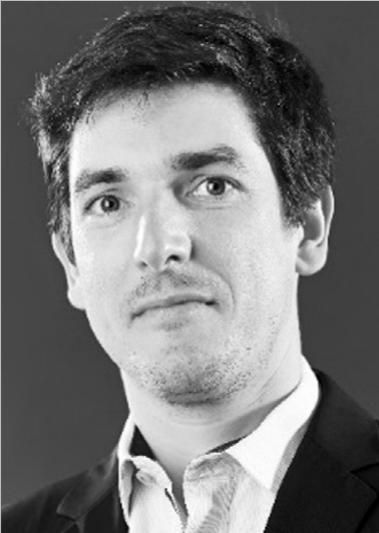

## Biographical Information

*Sebastian Henke studied Chemistry at the Ruhr‐University in Bochum (Germany), where he completed his PhD in Inorganic Chemistry with Roland A. Fischer in 2011. He then joined the lab of Sir Anthony K. Cheetham at the Department of Materials Science & Metallurgy of the University of Cambridge (UK). After a short stint in industry, he joined the Department of Chemistry and Chemical Biology at Technische Universität Dortmund as Junior Professor for Materials Chemistry in 2016. His research is focused on stimuli‐responsive MOFs and MOF glasses*.

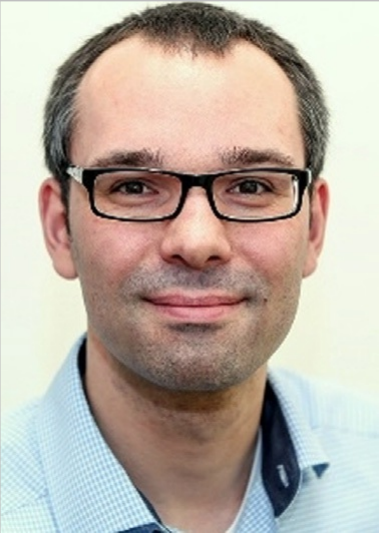

## Biographical Information

*Alexander Knebel studied Chemistry at the Leibniz University Hanover (Germany) and received his PhD in Physical Chemistry with Jürgen Caro in 2018. He conducted research with Jorge Gascon at the KAUST Catalysis Centre of the King Abdullah University of Science Technology (Saudi Arabia). Afterwards he was a postdoctoral fellow at the Institute of Functional Interfaces (IFG) with Christof Wöll at the Karlsruhe Institute of Technology (KIT) (Germany), where he recently became Junior Research Group Leader. In his research, he combines MOFs, COFs, porous liquids, and polymer mixed‐matrix systems in thin‐film gas‐separation membranes*.